# Supplementary material for: Chemical signatures of social information in Barbary macaques
Source: Sci Rep. 2025 Jan 14;15:1902. doi: 10.1038/s41598-024-84619-2 (PMC11730623; doi:10.1038/s41598-024-84619-2)
Supplement: Supplementary file 1 — Supplementary Material 1 [file 41598_2024_84619_MOESM1_ESM.pdf]

Chemical signatures of social information in Barbary macaques (Weiß et al.):

Supplemental methods and results

Details on paternity analysis

The 144 sampled individuals included 28 offspring from cohorts 2017-2019 (for 22 of which we also collected odor samples), their mothers and potential sires. Because Barbary macaques may also reproduce with extra-group partners [1], we considered all males in the population that were alive and at least 4 years of age at the onset of the respective mating season as potential sires. For animals born in cohort 2017 we were able to obtain samples for 96 %, and for cohorts 2018-2019, 97 % of all potential sires.

For 27 individuals blood samples had been collected during medical interventions prior to the onset of the study. Similarly, 7 tissue samples had previously been collected during medical intervention or from freshly deceased bodies. In recent years, hair samples have been routinely collected during tattooing of yearlings, so that hair samples were available for 68 of the individuals. To obtain DNA from 41 potential sires and one mother for whom no other sample types were available, we collected three fecal samples per individual immediately after defecation. Fecal samples were stored with the two-step alcohol-silica storage protocol [2]. DNA was extracted using DNEasy Blood & Tissue kit (Qiagen) for blood and tissue, and the First-DNA all-tissue DNA-Kit (GEN-IAL) for hair and feces.

We genotyped the DNA samples using a panel of up to 23 polymorphic microsatellite markers previously established for the study population [3], whereby we eventually focused on the 16 markers with the lowest dropout rate, that were most reliable, scorable and combinable in polymerase chain reactions (PCR, see Table S1). To increase the amount of host DNA in the samples we used the two-step multiplex approach for PCR amplification [4] by running a first PCR with all markers simultaneously, followed by a second set of PCRs containing diluted multiplex products as amplification templates and 4 – 6 fluorescently labelled primers with different allele ranges. PCRs were conducted in a Mastercycler® vapo.protect thermal cycler (Eppendorf, Hamburg, Germany) following the protocols of Westphal [3]. We conducted 2 independent sets of PCRs for each of the blood and tissue samples, and 3 for each of the hair and fecal samples. Due to the small amount of DNA and a high level of allelic dropouts in fecal samples [5], we analyzed two independent fecal samples per individual [e.g. 6].

We performed fragment analysis on an *ABI 3730* sequencer. For allele calling we used the Peak Scanner 2 software (Applied Biosystems®). For all sample types we confirmed heterozygous genotypes with two amplifications. For blood and tissue samples we confirmed homozygous genotypes with two, for hair samples with three and for fecal samples with four amplifications. In case of inconsistent results an additional sample of the respective individual was analyzed.

For 27 of the 28 offspring born 2017 – 2019 the observed mothers showed no mismatches to their offspring. For one offspring we could not confirm the observed mother genetically, probably due to poor sample quality. In this case we considered the observed mother to be the biological mother, but excluded the offspring from paternity analysis. We could also genetically confirm the observed mother for 38 females and males initially sampled as mothers or potential sires.

We used confirmed maternities in the paternity analysis, applying a combination of exclusion and likelihood methods for mother-offspring-putative father trios with the programs Findsire and Cervus following established criteria [e.g. 6,7]. In particular, we used the following exclusion criteria for paternity assignment with Findsire: the strict criterion was met if a sire showed no mismatches and the next potential sire at least two and the relaxed criterion if a sire showed no mismatches and the next potential sire one mismatch. Any cases in which all potential sires had at least one mismatch were considered unsolved. Cases in which two potential sires showed no mismatch were considered a tie. Ties were also considered unsolved unless one of the potential sires was assigned a likelihood of 95% in Cervus, in which case the sire designated by Cervus was accepted as the sire. In case of inconsistent assignments by Findsire and Cervus, paternity was considered unsolved.

We were able to assign fathers to 23 of the 28 offspring from cohorts 2017-2019 with high certainty. Furthermore, we accepted fathers assigned with high confidence for 18 individuals initially sampled as mothers or potential sires in the present study and born between 2011 and 2016. For these cohorts, 58-87 % of potential sires were genotyped. We did not accept fathers assigned to older individuals due to the increasingly lower proportion of potential sires sampled.

As input we used genetically confirmed mothers whenever available, and observed mothers (based on observational records from the field) for all remaining subjects, as well as all available paternity data. Given that mother-offspring dyads are available from long-term observational records, our value of pedigree relatedness for maternal relatedness can be considered as highly precise, reflecting close maternal ($r > 0.125$) but also distant maternal kin ($r \leq 0.125$), while our values of pedigree relatedness for the paternal line are likely underestimated in this analysis.

Table S1: Final microsatellite marker set used for parentage analysis. Primers in the same set were combined in the second PCR reaction.

**Primer**	**tandem repeat**	**labeled**	**allele size range**	**primer set**
D10s1432	tetra	hex	140-164	1
D1s548	tetra	hex	184-208	1
D2s1333	tetra	hex	290-306	1
D12s67	tetra	fam	126-142	1
D6s493	tetra	fam	154-166	1
D13s765	tetra	fam	177-194	1
D10s611	tetra	hex	157-185	2
D19s245	tetra	hex	249-333	2
D19s582	tetra	fam	131-143	2
D4s243	tetra	fam	191-203	2
D7s2204	tetra	ned	227-243	2
D5s1467	tetra	hex	192-200	3
D5s1466	tetra	hex	273-329	3
D7s503	di	fam	142-164	3
D1s518	tetra	fam	186-206	3
D11s925	di	ned	189-205	3

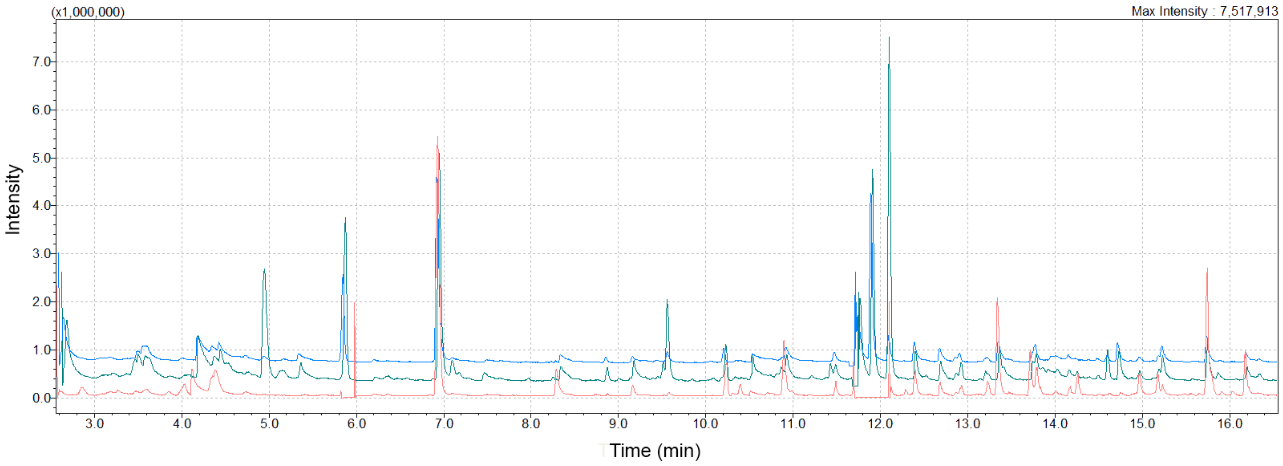

Figure S1: Chromatograms of Barbary macaque body odor samples from two different females (blue and green lines) and a blank control sample (orange line) over the volatile range. Base lines were slightly shifted along the y-axis for better visibility of the individual lines.

Table S2: List of focal individuals, their identity (IDcode), sex, year of birth (cohort) and group membership. "contraception" denotes for all adult females if the individual received hormonal contraception during the study period. "rank_cat", "matkin_unit" and "patkin_unit" denote the dominance category and family unit membership for those of the focal individuals used in the respective analysis.

IDcode	sex	cohort	group	contraception	rank_cat	matkin_unit	patkin_unit
ID01	male	2017	H	NA	NA	m7	p5
ID02	male	2009	C	NA	NA	NA	NA
ID03	male	2010	H	NA	low	NA	NA
ID04	female	2018	F	no	NA	m10	p9
ID05	male	2004	C	NA	high	NA	p4
ID06	male	2007	H	NA	high	NA	NA
ID07	female	2017	F	no/yes	low	NA	NA
ID08	male	2006	H	NA	NA	NA	p7
ID09	male	2009	H	NA	low	m6	NA
ID10	female	2012	H	yes	NA	NA	NA
ID11	male	2009	C	NA	NA	NA	p3
ID12	female	2011	C	yes	high	m11	NA
ID13	female	2012	H	no	high	m7	NA
ID14	male	2011	F	NA	NA	NA	p2
ID15	female	2017	F	no	NA	NA	p1
ID16	female	2002	H	no	NA	NA	NA
ID17	female	2011	C	yes	NA	m4	NA
ID18	female	2017	C	no	NA	m4	p12
ID19	female	2009	F	yes	high	NA	NA
ID20	female	2012	F	yes	low	m2	NA
ID21	male	2008	H	NA	NA	NA	NA
ID22	male	2004	C	NA	NA	NA	p12
ID23	male	2017	F	NA	NA	m5	p2
ID24	female	2012	F	yes	NA	m5	NA
ID25	male	1999	H	NA	high	NA	NA
ID26	female	2018	F	no	NA	m8	NA
ID27	male	2000	F	NA	NA	NA	p1
ID28	male	2007	C	NA	low	NA	NA
ID29	female	2019	C	no	NA	m12	p12
ID30	male	2009	C	NA	high	NA	NA
ID31	male	2006	F	NA	NA	NA	p11
ID32	male	2004	H	NA	NA	NA	p5
ID33	male	2019	F	NA	NA	m9	p6
ID34	female	2006	F	yes	high	m1	NA
ID35	male	2008	F	NA	high	NA	p10
ID36	male	2013	F	NA	low	NA	p6
ID37	male	2009	C	NA	low	NA	p8
ID38	female	2018	H	no	NA	NA	p5
ID39	female	2019	C	no	NA	m3	p3
ID40	male	2013	C	NA	low	NA	NA
ID41	male	2018	H	NA	NA	m6	NA
ID42	female	2007	F	yes	NA	NA	NA
ID43	male	2003	C	NA	high	NA	NA
ID44	male	2008	F	NA	high	NA	NA

ID45	female	2014	C	yes	high	m12	p12
ID46	female	2005	H	yes	low	m6	NA
ID47	male	2017	H	NA	NA	NA	NA
ID48	female	2008	C	yes/no	low	m10	NA
ID49	male	2019	F	NA	NA	m1	p11
ID50	male	2008	C	NA	low	NA	NA
ID51	female	2014	C	yes	high	m3	NA
ID52	male	1995	C	NA	NA	NA	NA
ID53	male	1999	F	NA	NA	NA	NA
ID54	male	2007	H	NA	NA	NA	NA
ID55	female	2013	F	yes	low	m10	NA
ID56	male	2018	C	NA	NA	m11	p4
ID57	female	2011	F	yes	high	m1	NA
ID58	female	2015	F	yes	high	m1	p8
ID59	female	2017	F	no	NA	NA	p10
ID60	female	2005	F	yes	NA	NA	NA
ID61	male	2017	C	NA	NA	m11	p3
ID62	female	2014	C	no	low	NA	NA
ID63	male	2018	H	NA	NA	NA	p7
ID64	female	2017	F	no	low	m2	p2
ID65	male	2009	F	NA	low	NA	NA
ID66	male	2018	H	NA	NA	m7	NA
ID67	male	2012	F	NA	high	NA	NA
ID68	female	2013	F	yes	NA	m8	NA
ID69	female	2008	H	yes	high	m7	NA
ID70	female	2014	F	yes	low	m9	NA
ID71	male	2013	F	NA	low	NA	p9
ID72	female	2017	C	no	NA	NA	NA

Detailed results tables

Table S3: Detailed results of perMANOVAs conducted with data subsets for ID, kinship and dominance class.

subset	term	Df	SumOfSqs	R2	F	P
	ID	21	12.084	0.203	3.838	0.001
	sex	0	0.000	0	Inf	
	age	1	7.620	0.128	50.823	0.001
ID	group	1	0.072	0.001	0.478	0.989
	season	2	8.085	0.136	26.961	0.001
	residual	214	32.087	0.538		
	total	241	59.628	1		
	kin unit	11	5.27	0.099	2.537	0.001
	sex	1	0.276	0.005	1.461	0.001
maternal kin	season	2	7.74	0.145	20.493	0.001
	age	1	0.47	0.009	2.488	0.001
	residual	204	38.523	0.72		
	total	219	53.479	1		
	kin unit	11	5.877	0.132	2.772	0.067
	sex	1	0.201	0.005	1.043	0.001
paternal kin	season	2	4.956	0.111	12.855	0.001
	age	1	0.340	0.008	1.761	0.790
	residual	163	31.419	0.706		
	total	178	44.528	1		
	sex	1	1.254	0.019	6.535	0.001
	dominance class	1	0.169	0.003	0.879	0.008
	age	1	0.769	0.012	4.007	0.001
dominance class	group	2	3.063	0.047	7.982	0.026
	season	2	8.856	0.136	23.080	0.001
	residual	252	48.345	0.742		
	total	259	65.153	1		

Table S4: Estimates for all fixed and random terms from the multi-membership model on genetic relatedness rank. CrI = Credible Interval. ESS = effective sample size. "d_xxx" denotes delta, i.e. the difference of the respective term between the two samples in a given dyad.

**fixed term**	**Estimate**	**Est.Error**	**2.5% CrI**	**97.5% CrI**	**Rhat**	**bulk ESS**	**tail ESS**
Intercept	0.142	0.026	0.089	0.194	1.002	1592	2884
maternal r	0.000	0.001	-0.002	0.002	1.000	9718	7456
paternal/mixed r	-0.002	0.002	-0.005	0.001	1.000	6365	5522
d_elo score	0.000	0.001	-0.002	0.002	1.000	10551	7505
d_sex (same)	-0.002	0.002	-0.005	0.002	1.000	9026	7614
d_age	-0.001	0.002	-0.004	0.002	1.000	6827	6459
d_group (same)	0.012	0.006	0.001	0.023	1.001	2163	3956
d_season (same)	-0.024	0.012	-0.048	-0.001	1.003	562	1399
d_date	-0.320	0.009	-0.339	-0.301	1.007	528	944
d_time	0.001	0.001	-0.002	0.003	1.000	4987	6121
d_bodyregion (same)	0.003	0.003	-0.003	0.008	1.001	4418	5675
d_s.distance	0.004	0.002	0.000	0.009	1.001	3783	5261
d_s.volume	0.007	0.005	-0.004	0.016	1.003	984	1902
d_subsamples	-0.007	0.002	-0.011	-0.003	1.001	4902	6101
d_batch (same)	0.199	0.015	0.169	0.230	1.001	1051	1941
**random term**	**Estimate**	**Est.Error**	**2.5% CrI**	**97.5% CrI**	**Rhat**	**bulk ESS**	**tail ESS**
sample (Intercept)	0.399	0.014	0.372	0.429	1.003	1074	2166
ID (Intercept)	0.149	0.028	0.096	0.204	1.003	471	973
ID (maternal r)	0.003	0.002	0.000	0.007	1.001	1781	3738
ID (paternal/mixed r)	0.007	0.002	0.003	0.012	1.002	1739	1761
ID (d_elo score)	0.003	0.002	0.000	0.007	1.000	1876	3449
ID (d_sex)	0.003	0.003	0.000	0.009	1.000	2608	3813
ID (d_age)	0.009	0.002	0.005	0.013	1.000	2379	2364
ID (d_group)	0.043	0.005	0.035	0.053	1.000	3230	5042
ID (d_season)	0.094	0.009	0.078	0.113	1.002	1607	3186
ID (d_date)	0.081	0.007	0.068	0.097	1.002	913	2059
ID (d_time)	0.009	0.001	0.006	0.012	1.001	3418	5151
ID (d_bodyregion)	0.019	0.003	0.014	0.026	1.000	3248	4049
ID (d_s.distance)	0.016	0.002	0.012	0.020	1.000	3528	5360
ID (d_s.volume)	0.041	0.004	0.034	0.049	1.000	1500	3193
ID (d_subsamples)	0.013	0.002	0.010	0.018	1.000	3736	5669
ID (d_batch)	0.124	0.012	0.103	0.149	1.001	2176	4369
dyad (Intercept)	0.019	0.001	0.016	0.021	1.001	1690	3502
dyad (d_season)	0.040	0.002	0.036	0.044	1.000	2014	4321
dyad (d_date)	0.005	0.002	0.000	0.009	1.012	462	1413
dyad (d_time)	0.007	0.002	0.003	0.009	1.003	902	634
dyad (d_bodyregion)	0.029	0.002	0.025	0.032	1.001	2629	4323
dyad (d_s.distance)	0.017	0.001	0.015	0.019	1.001	2302	4114
dyad (d_s.volume)	0.012	0.001	0.009	0.014	1.002	1642	2844
dyad (d_subsamples)	0.016	0.001	0.014	0.018	1.002	2199	4233
dyad (d_batch)	0.087	0.003	0.081	0.093	1.000	2602	5162

Table S5: Estimates for all fixed and random terms from the multi-membership model on rank. CrI = Credible Interval. ESS = effective sample size. “d_xxx” denotes delta, i.e. the difference of the respective term between the two samples in a given dyad.

**fixed term**	**Estimate**	**Est.Error**	**2.5% CrI**	**97.5% CrI**	**Rhat**	**bulk ESS**	**tail ESS**
Intercept	0.154	0.039	0.076	0.232	1.001	2680	5074
d_elo score	0.000	0.001	-0.003	0.003	1.000	19102	9841
total r	-0.002	0.001	-0.005	0.001	1.000	18584	8977
sex (male)	-0.023	0.053	-0.126	0.080	1.001	2879	5192
d_age	0.001	0.002	-0.003	0.005	1.000	14446	9510
d_group (same)	0.016	0.007	0.002	0.030	1.000	5497	7723
d_season (same)	-0.027	0.011	-0.048	-0.006	1.002	2736	4694
d_date	-0.325	0.010	-0.346	-0.305	1.002	1088	2508
d_time	0.001	0.001	-0.002	0.003	1.000	15821	9039
d_bodyregion (same)	0.004	0.003	-0.002	0.011	1.000	11941	8932
d_s.distance	0.005	0.002	0.001	0.010	1.001	11074	9460
d_s.volume	0.008	0.005	-0.003	0.019	1.001	2684	3564
d_subsamples	-0.007	0.002	-0.011	-0.003	1.000	15094	10231
d_batch (same)	0.184	0.018	0.148	0.219	1.001	2652	5513
**random term**	**Estimate**	**Est.Error**	**2.5% CrI**	**97.5% CrI**	**Rhat**	**bulk ESS**	**tail ESS**
sample (Intercept)	0.395	0.014	0.368	0.424	1.002	2096	4665
ID (Intercept)	0.151	0.028	0.099	0.208	1.006	891	1598
ID (d_elo score)	0.003	0.002	0.000	0.008	1.002	3549	6077
ID (total r)	0.003	0.002	0.000	0.009	1.001	3539	5713
ID (d_age)	0.010	0.003	0.004	0.016	1.001	2014	1208
ID (d_group)	0.053	0.007	0.041	0.067	1.001	5572	8358
ID (d_season)	0.089	0.009	0.074	0.108	1.001	4696	7678
ID (d_date)	0.086	0.008	0.072	0.103	1.001	2359	4170
ID (d_time)	0.007	0.002	0.002	0.011	1.001	2548	2941
ID (d_bodyregion)	0.021	0.004	0.013	0.030	1.000	4347	5327
ID (d_s.distance)	0.016	0.003	0.011	0.022	1.001	5362	7222
ID (d_s.volume)	0.042	0.004	0.035	0.051	1.001	5052	7551
ID (d_subsamples)	0.011	0.003	0.005	0.017	1.001	3055	3489
ID (d_batch)	0.145	0.017	0.115	0.182	1.000	4540	6374
dyad (Intercept)	0.019	0.002	0.015	0.023	1.002	2254	4976
dyad (d_season)	0.041	0.003	0.036	0.046	1.001	3907	6449
dyad (d_date)	0.006	0.003	0.000	0.012	1.007	872	2856
dyad (d_time)	0.006	0.002	0.001	0.010	1.006	1306	1690
dyad (d_bodyregion)	0.031	0.002	0.026	0.036	1.000	4125	6872
dyad (d_s.distance)	0.018	0.001	0.015	0.021	1.001	3782	7017
dyad (d_s.volume)	0.012	0.002	0.008	0.015	1.001	2756	3704
dyad (d_subsamples)	0.015	0.002	0.012	0.019	1.002	2898	4596
dyad (d_batch)	0.086	0.004	0.078	0.095	1.000	5176	8510

Table S6: Estimates for all fixed and random terms for the compositional model on social attributes. CrI = Credible Interval. ESS = effective sample size.

**fixed term**	**Estimate**	**Est.Error**	**2.5% CrI**	**97.5% CrI**	**Rhat**	**bulk ESS**	**tail ESS**
Intercept	-0.003	0.164	-0.326	0.321	1.002	2990	4034
elo score	0.000	0.009	-0.018	0.018	1.001	10948	6198
sex (male)	0.000	0.022	-0.042	0.043	1.000	9709	5772
age	0.000	0.011	-0.021	0.021	1.000	10765	6682
group (F)	0.000	0.038	-0.073	0.073	1.000	6257	5939
group (H)	0.001	0.038	-0.074	0.075	1.000	5793	5660
season (spring)	0.002	0.114	-0.225	0.225	1.000	3150	4068
season (summer)	0.001	0.113	-0.223	0.224	1.001	3400	4811
time	0.000	0.009	-0.018	0.018	1.001	11210	6215
bodyregion (lower)	-0.001	0.094	-0.182	0.182	1.000	5589	5075
bodyregion (mixed)	0.000	0.093	-0.180	0.183	1.000	5615	5251
bodyregion (upper)	-0.001	0.095	-0.186	0.183	1.000	5692	5283
s.distance	0.000	0.023	-0.045	0.044	1.001	2774	3951
s.volume	0.000	0.060	-0.118	0.116	1.006	539	1202
subsamples	0.000	0.015	-0.028	0.029	1.000	7401	6173
**random term**	**Estimate**	**Est.Error**	**2.5% CrI**	**97.5% CrI**	**Rhat**	**bulk ESS**	**tail ESS**
sample (Intercept)	0.007	0.005	0.000	0.019	1.001	5732	3816
compound (Intercept)	1.500	0.096	1.320	1.695	1.003	2217	3112
compound (elo score)	0.017	0.012	0.001	0.043	1.002	3304	3855
compound (sex)	0.102	0.019	0.063	0.138	1.001	2819	3082
compound (age)	0.052	0.017	0.012	0.083	1.002	1709	1218
compound (group)	0.290	0.016	0.261	0.321	1.001	3772	4889
compound (season)	1.060	0.042	0.982	1.146	1.002	2698	4371
compound (time)	0.040	0.018	0.004	0.072	1.003	1657	2624
compound (bodyregion)	0.133	0.018	0.099	0.167	1.001	2690	3865
compound (s.distance)	0.262	0.018	0.229	0.298	1.000	2989	5028
compound (s.volume)	0.810	0.042	0.734	0.899	1.002	1140	2388
compound (subsamples)	0.128	0.015	0.099	0.157	1.000	3441	5449
ID (Intercept)	0.007	0.005	0.000	0.020	1.000	5272	3378
ID (season)	0.007	0.005	0.000	0.020	1.001	6584	4186
ID (time)	0.007	0.005	0.000	0.019	1.001	6170	3882
ID (bodyregion)	0.007	0.005	0.000	0.019	1.001	5767	3616
ID (s.distance)	0.007	0.005	0.000	0.020	1.000	6069	3831
ID (s.volume)	0.007	0.005	0.000	0.020	1.000	5974	3933
ID (subsamples)	0.007	0.006	0.000	0.021	1.001	5094	3889
date (Intercept)	0.007	0.005	0.000	0.020	1.000	5750	3828
date (elo score)	0.007	0.005	0.000	0.019	1.000	6316	3602
date (sex)	0.007	0.005	0.000	0.019	1.000	5381	3579
date (age)	0.007	0.005	0.000	0.019	1.000	5632	3566
date (group)	0.007	0.005	0.000	0.019	1.000	5834	3434
date (time)	0.007	0.005	0.000	0.019	1.000	5882	3499
date (bodyregion)	0.007	0.005	0.000	0.019	1.000	5275	3463
date (s.distance)	0.007	0.005	0.000	0.019	1.001	5856	3897
date (s.volume)	0.007	0.005	0.000	0.020	1.000	6369	4406
date (subsamples)	0.007	0.005	0.000	0.020	1.000	5913	3819
batch (Intercept)	0.011	0.009	0.000	0.035	1.000	5531	4316
tube (Intercept)	0.007	0.005	0.000	0.019	1.000	5914	3912

Table S7: List of compounds potentially associated with sex and age differences in chemical profiles of Barbary macaque samples. Credible intervals of compounds listed in association with sex overlapped between the sexes, but the estimate (intercept) for one sex was outside the credible interval of the other. Similarly, credible intervals of compounds listed in association with age overlapped 0, but did so by < 20 % of the respective credible interval. RT = retention time (in min.), base peak describes the largest mass fragment of the peak. SI = similarity index (from 0 - 100) for the match between mass spectra and their respective best hit in the NIST library.

**trait**	**RT**	**base peak**	**best NIST library hit**	**SI**	**substance class**	**found in**
age	4.44	59	Formamide, N-methyl-	95	amide	humans [8]
age	6.36	91	Ethylbenzene	85	aromatic hydrocarbon	humans [8–10]
sex	9.25	94	Undecanal	91	aldehyde	humans [8,11,12], mandrills [13]
sex	10.07	93	3-Carene	92	monoterpene	human excretions [9]
sex	14.19	67	unidentified	< 80		
age	17.73	85	unidentified	< 80		
sex	30.84	119	unidentified	< 80		
sex	31.34	119	unidentified aldehyde	< 80	aldehyde	
sex	44.50	103	Unidentified ester	< 80	ester	

Additional references:

1. Brauch, K. *et al.* Sex-specific reproductive behaviours and paternity in free-ranging Barbary macaques (*Macaca sylvanus*). *Behav. Ecol. Sociobiol.* **62**, 1453–1466 (2008).
2. Nsubuga, A. M. *et al.* Factors affecting the amount of genomic DNA extracted from ape faeces and the identification of an improved sample storage method. *Mol. Ecol.* **13**, 2089–2094 (2004).
3. Westphal, H. Paternity assessment in Barbary macaques. (Leipzig University, Leipzig, 2020).
4. Arandjelovic, M. *et al.* Two-step multiplex polymerase chain reaction improves the speed and accuracy of genotyping using DNA from noninvasive and museum samples. *Mol. Ecol. Resour.* **9**, 28–36 (2009).
5. Bayes, M. K., Smith, K. L., Alberts, S. C., Altmann, J. & Bruford, M. W. Testing the reliability of microsatellite typing from faecal DNA in the savannah baboon. *Conserv. Genet.* **1**, 173–176 (2000).
6. Minkner, M. M. I. *et al.* Assessment of male reproductive skew via highly polymorphic STR markers in wild vervet monkeys, *Chlorocebus pygerythrus*. *J. Hered.* **109**, 780–790 (2018).
7. Ruiz-Lambides, A. V., Weiß, B. M., Kulik, L. & Widdig, A. Which male and female characteristics influence the probability of extragroup paternities in rhesus macaques, *Macaca mulatta*? *Anim. Behav.* **140**, 119–127 (2018).
8. Costello, B. de L. *et al.* A review of the volatiles from the healthy human body. *J. Breath Res.* **8**, 014001 (2014).
9. Garner, C. E. *et al.* Volatile organic compounds from feces and their potential for diagnosis of gastrointestinal disease. *FASEB J.* **21**, 1675–1688 (2007).
10. Bernier, U. R., Kline, D. L., Barnard, D. R., Schreck, C. E. & Yost, R. A. Analysis of Human Skin Emanations by Gas Chromatography/Mass Spectrometry. 2. Identification of Volatile Compounds That Are Candidate Attractants for the Yellow Fever Mosquito (*Aedes aegypti*). *Anal. Chem.* **72**, 747–756 (2000).
11. Curran, A. M., Rabin, S. I., Prada, P. A. & Furton, K. G. Comparison of the volatile organic compounds present in human odor using spme-GC/MS. *J. Chem. Ecol.* **31**, 1607–1619 (2005).
12. Penn, D. J. *et al.* Individual and gender fingerprints in human body odour. *J. R. Soc. Interface* **4**, 331–340 (2007).
13. Vaglio, S. *et al.* Sternal Gland Scent-Marking Signals Sex, Age, Rank, and Group Identity in Captive Mandrills. *Chem. Senses* **41**, 177–186 (2016).
